# Unconnected and out-of-sight: identifying health care non-users with unmet needs

**DOI:** 10.1186/s12913-017-2019-4

**Published:** 2017-01-25

**Authors:** Elizabeth Hoon, Clarabelle Pham, Justin Beilby, Jonathan Karnon

**Affiliations:** 1 School of Public Health, Mail Drop DX 650 171, The University of Adelaide, Adelaide, SA 5005 Australia; 2 Torrens University, GPO Box 2025, Adelaide, SA 5001 Australia

**Keywords:** Non-users, Measurement of omission, Health service use

## Abstract

**Background:**

While current debates on how to deliver sustainable health care recognise socio-economic dimensions to health service use, attention has focussed on how to reduce demand for services. However, the measures of demand may not account for a subgroup of the population who to date have remained out of sight because they do not access health services. This study aimed to describe the characteristics of individuals who self-reported having fair or poor health but did not use health services.

**Methods:**

Data from the 2010 LINKIN health census survey (*n* = 7895) and the 2013 HILDA National Panel Survey (*n* = 13,609) were analysed focussing on the population who self-reported their overall health status as fair or poor. Simple and multivariable logistic regression modelling examined characteristics associated with a lack of health services use. The outcome measure of interest was no health service use in the previous 12 months and co-variables included demographic and socioeconomic indicators, health-related quality of life, having no health condition and health risk factors.

**Results:**

Overall 21% of LINKIN respondents reported their overall health as fair or poor compared to 18% in the HILDA dataset. In LINKIN, 4.4% of those reporting fair or poor health, reported not using any health service provider in the past 12 months. Similarly, 4.5% of HILDA respondents were non-users. When adjusted for multiple co-variables, unemployment (aOR 3.24, 95% CI 1.28-8.17), educational level at Year 10 or below (aOR 1.94, 95% CI 1.02-3.70) and smoking (aOR 2.67, 95% CI 1.38-5.17) were significantly associated with non-use for the LINKIN data, as did lack of health conditions (aOR 0.18, 95% CI 0.08-0.41). The HILDA regression analyses indicated the same directions of association between equivalent variables and lack of health service use, with the exception of educational level.

**Conclusions:**

In line with recent assertions on real denominators in health need, this study describes those people rarely included in the population at risk and the potential for systematic bias towards the overestimation of the effectiveness of interventions. This study informs current policy debates and planning, including how we connect with hard-to-reach populations and how this sub-group might be more appropriately included when measuring effectiveness of health policies and programs.

## Background

Current and projected patterns of health service use are the central tenets of the current debate on how to deliver sustainable models of care within an ever stretched health budget [1, 2]. While this debate has recognised that there are socio-economic dimensions to health service use, attention has focussed on how to reduce demand for services (e.g. by reducing preventable hospital visits, reducing over diagnosis and over treatment, and the need for a price signal for users of the system). Yet, a sub-group of the population that remains out of sight in this debate are individuals with health care needs who are unconnected with health services. Their very lack of health service use means that they remain beyond the scope of most health service analyses, policy and practice development.

The measurement of omission has been identified as a concern for modern health systems where it is acknowledged that simple notions of ‘access’ are not enough to address gaps in coverage [3]. Without the identification of patients not included, treated, or followed up, the inverse care law [4] is likely to remain or become wider; that is, the organisation and delivery of services becomes itself part of the social determinants of health. Indeed, in championing ‘real’ evidence based medicine, Greenhalgh and colleagues have recently argued that the ‘hidden denominator’ of people who do not seek or cannot access care is a source of systematic bias when measuring the true effectiveness of evidence based medicine [5, 6].

To date this population has not been well described within an Australian setting, with no Australian data available for inclusion in a recent international scoping review, profiling attendees and non-attendees of health checks in primary health [7]. This scoping review [7] found that people who did not attend health checks were more likely to be male, on low incomes, have low socio-economic status and be less well educated. Marital status was a factor with non-attenders more likely to be single. This international scoping review (including studies from North America (*n* = 13), Europe (*n* = 24) Israel (*n* = 1) and Taiwan (*n* = 1)) also identified that non-attenders of health checks were more likely to have cardiovascular risk factors including cigarette smoking [7].

This paper examines the characteristics associated with a lack of health services use (in the past 12 months) in a sub-group reporting fair or poor health (as measured by the Short Form 1 (SF-1)) within an Australian health system setting. The purpose of this study is to inform current policy debates and planning, including how primary health care providers connect with underserved populations and how this sub-group might be more appropriately included when measuring the effectiveness of health policies and programs.

## Methods

### Population

**Fig. 1:**
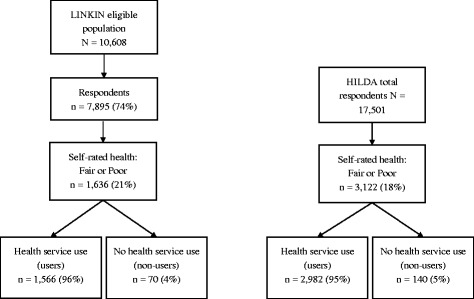
Flowchart for LINKIN and HILDA datasets

This study used two datasets; the 2010 health census of the Port Lincoln community which was part of the LINKIN Health Study [8]; and the Household, Income and Labour Dynamics in Australia (HILDA) National Panel Survey (Wave 13, data collected in 2013) [9] (Fig. 1). HILDA is a continuing nationally representative longitudinal survey of Australian households with an interest on family and household formation, income and work. While most questions in the survey are repeated annually, in Waves 9 and 13 a module of questions focussed on health were included. The HILDA sample selection was stratified by State, and within the five largest States in terms of population, by metropolitan and non-metropolitan regions [9]. To adjust for differential unit non-response experienced at both the household and person-level, and to account for unequal probabilities of selection into the HILDA sample, cross sectional weights (using the Replicate Weight Method) were used for all analyses of HILDA data [10].

The LINKIN Health Study was conducted in Port Lincoln, a regional centre in South Australia with an eligible population (aged 15 years and over) of 10,608. A key aim of the health census was to collect data from the population of this distinct bounded locale, not just those who were connected to programs and services (*n* = 7895, response rate, 74%). This health census included non-private households (e.g. nursing homes, shared community supported households, shelters, and households living at the local caravan park for 6 weeks or more) as well as private households. Its methodology is described in more detail elsewhere [8], but briefly, the design and implementation differed somewhat from the mandatory Australian Government Census as it required a greater number of delivery and collection attempts with a specific focus on face-to-face contact. A minimum of three attempts to deliver and three attempts to collect the census questionnaires were made at each household with self-addressed envelopes left for postal return if face-to-face contact was not established. These delivery and collection attempts were spaced apart to include day-time, evening and weekend visits to ensure maximum opportunity for face-to-face contact. This intensive approach gave an opportunity to explain the health census and to support participation through assistance in completing the questionnaires when required, and resulted in markedly improved response rates (89% participation when a face-to-face delivery was achieved compared to 26% participation when no contact was made with householders). Data was collected on socio-demographic characteristics, current medical conditions, health-related quality of life and health service use using self-completion household and individual questionnaires for all residents (aged 15 years and over). A modified household questionnaire was used to collect data for the non-private households. Ethics approval for the study was obtained from the University of Adelaide (H-036-2010). The LINKIN Health study anonymised dataset may be accessed by researchers for analysis purposes upon request to the first author. Access to the HILDA datasets is at the discretion of the Longitudinal Surveys Business Owner, a position held by the Branch Manager of the National Centre for Longitudinal Data, Policy Evidence Branch in the Department of Social Services (DSS), Australia. A license application to use may be accessed via the HILDA home webpage.

### Measures

The SF-1 [11] is a measure of overall health status and was used as an indicator of potential health service need. It has been demonstrated to be a strong indicator of future health care use and mortality [12, 13]. Respondents were asked, “In general would you say that your health is: Excellent, Very Good, Good, Fair or Poor?” Those who reported fair or poor health were included in this analysis.

In the LINKIN health census, non-use of health services was defined using the question; ‘Please indicate, as best you can, the number of visits you have made to the following health services in the LAST 12 MONTHS?’. A broad range of nineteen health services were included in the associated response table and included the Aboriginal health service, hospital as an in-patient, hospital as an outpatient, GP during hours, GP out of hours, practice nurse, allied health professionals, specialists, and alternative health providers. In order to reflect service access issues in regional Australia, a distinction between providers in Port Lincoln and elsewhere was made. Respondents, who reported making no visits to providers in Port Lincoln or elsewhere, were identified as non-users.

In order to gain a detailed understanding of this population’s characteristics compared to people with fair or poor health who used health services, descriptive analysis identified variables that could potentially influence health service use [7]. The variables were grouped into four domains: demographic (sex (male versus female), age (continuous variable), marital status (not single versus single/ never married)); socio-economic position (education level (Year 11 or above versus Year 10 or below), employment status (not unemployed versus unemployed); quality of life (no problem versus at least some problems for each of the EuroQol five dimensions three level questionnaire **(**EQ-5D-3L) [14]); health conditions (none versus one or more) and smoking status (not a current smoker versus current smoker). The first response for all the variables was used as the reference category for all analyses.

HILDA data was used to assess the generalizability of the results from the LINKIN population across a broader sample. HILDA was preferred over other large national datasets as its primary focus was not health, thereby broadening response appeal and sample retention to those not engaged as well as those engaged with the topic of ‘health’. People reporting fair or poor health were identified using the same general health question within the 6-Item Short Form Health Survey (SF-36 scale) used in HILDA. Non-users were identified as those who had not visited a general practitioner or any other health service providers in the past 12 months (identified from a list supplied by the interviewer). Equivalent socio demographic variables to the LINKIN dataset were used with the exception of the health related quality of life variables. The EQ-5D questions were not included in the HILDA survey and therefore a comparative analysis (including multivariable logistic regression modelling) was not extended to health-related quality of life. The HILDA dataset was also examined by residential location of respondent as it was acknowledged that LINKIN data was collected from a regional Australian community. A dichotomous variable that identified whether HILDA respondents lived in a city or not (i.e. in an inner or outer regional, remote or very remote area, derived from the Accessibility / Remoteness Index of Australia (ARIA) scores) [15] was used to conduct some specific analyses for those living in a non-city location.

### Data analyses

Simple logistic regression compared proportions of participants with fair or poor health, who were ‘users’ and ‘non-users’ of health services with each of the variables outlined above. Multivariable logistic regression modelling [16] was then used to adjust for differences in the underlying characteristics of user and non-user respondents. Independent variables included in this multivariable modelling were those with a p-value below 0.3 in the simple logistic regression or were identified from existing literature (as in the case of gender for the HILDA dataset) as potentially influential of health service usage [7]. All models were assessed for multicollinearity [17]. Analyses were performed using Stata, release 14.0 (Statacorp LP). Results were presented as adjusted odds ratios (aORs) with 95% confidence intervals (CI). Separate analyses were undertaken for the LINKIN and the HILDA data. The simple logistic regression analyses of the LINKIN data included health-related quality of life, whilst the HILDA analyses investigated differences between metropolitan and non-metropolitan areas.

## Results

Overall 21% of LINKIN respondents (*n* = 1636) reported their overall health as fair or poor compared to 18% in the HILDA dataset (*n* = 3122). In LINKIN, 4.4% (*n* = 70) of those reporting fair or poor health, reported not using any health service provider in the past 12 months. Similarly, 4.5% (*n* = 140) of HILDA respondents were non-users.

Using simple logistic regression both datasets indicated the same directions of association for all co variables with the exception of educational level. Compared to the LINKIN population who reported fair or poor health and use of health services, non-users were more likely to be younger (OR 0.98, 95% CI 0.97-0.99), single or never married (OR 2.50, 95% CI 1.44-4.34), daily or occasional smokers (OR 3.81, 95% CI 2.26-6.40), and unemployed (OR 4.89, 95% CI 2.44-9.82) (Table 1). A higher proportion of non-users reported no health conditions (24% compared to 5% in health services users) (Table 1); although non-users without a reported condition were more likely than users without a health condition to have at least one problem in one or more of the EQ-5D dimensions of health. Further, non-users were less likely to report problems with mobility, pain or discomfort and usual activities, but more likely to report at least moderate anxiety/ depression (Table 1). The HILDA data showed the same directions of association for the co-variables related to age, gender, marital status, health conditions (all significant at 95% CI), smoking and employment status (both not significant at 95% CI). Of note, the HILDA data showed a stronger result for gender, with non-users more likely to be male (OR 1.65, 95% CI 0.95-2.85). As mentioned previously, the association between educational level and lack of health service use differed between the two datasets, with HILDA data indicating that non users were more likely to report an educational level of Year 11 or above and non-users in the LINKIN data more likely to report an educational level of Year 10 or below. It should be noted that for LINKIN the education effect was non-significant but for HILDA it was significant (Table 1). Further, when the HILDA data was examined by geographic location, non-users in non-metropolitan regions were still more likely to report education level of Year 11 or above, the same sign as the overall HILDA sample, but was not significant (Table 1).

**Table 1:** Characteristics of the population who reported fair or poor health and whether they had used at least one health service in the past 12 months

	LINKIN (*n* = 1630)				HILDA Non-metropolitan* (*n* = 1300)				HILDA* (*n* = 3122)			
	Non-users, n (%)	OR	95% CI	*P*	Non-users %	OR	95% CI	*P*	Non-users %	OR	95% CI	*P*
Fair or poor health	70 (4.4)				68 (5.2)				(140) 4.5			
Mean age (SD) (years)	56.2 (18.2)	0.98	0.97-0.99	0.013	39.7 (13.1)	0.95	0.94-0.97	<0.001	40.8 (15.3)	0.96	0.95- 0.97	<0.001
Sex												
Males	38 (55.1)				68.2				65.0			
Females	31 (44.9)	0.65	0.40-1.06	0.085	31.8	0.49	0.25-0.97	0.04	35.0	0.52	0.29-0.94	0.031
Marital status												
Not ‘single/never married’	49 (72.1)				69.7				58.4			
Single/never married	19 (27.9)	2.5	1.44- 4.34	<0.001	30.3	1.68	0.87-3.24	0.12	41.6	2.9	1.63-3.33	0.001
Smoking status												
Not a smoker	27 (44.3)				46.4				60.9			
Smoker	34 (55.7)	3.81	2.26- 6.40	<0.001	53.6	3.29	1.61-6.73	0.002	39.1	2.08	0.97-4.47	0.06
Work status												
Not unemployed	54 (83.1)				91.3				86.2			
Unemployed	11 (16.9)	4.89	2.44 -9.82	<0.001	8.8	2.51	0.63-9.93	0.186	13.8	4.07	0.71-28.1	0.108
Highest qualification												
> Year 11 or above	19 (30.2)				53.5				71.8			
Year 10 or below	44 (69.8)	1.65	0.95-2.85	0.075	46.5	0.86	0.48-1.5	0.598	28.2	0.59	0.36-0.90	0.017
Number of health conditions												
0	17 (24.3)				50.3				65.3			
1 or more	53 (75.7)	0.16	0.09- 0.29	<0.001	49.7	0.24	0.13-0.46	<0.001	34.7	0.14	0.08-0.25	<0.001
EQ-5D Anxiety/ depression												
no problems	18 (31.6)											
At least some	39 (68.4)	1.9	1.08- 3.35	0.027								
EQ-5D Mobility												
no problems	34 (56.7)											
At least some	26 (43.3)	0.76	0.45- 1.28	0. 303								
EQ-5D Pain/discomfort												
no problems	18 (29.5)											
At least some	43 (70.5)	0.57	0.33- 1.01	0.052								
EQ-5D Usual activities												
no problems	24 (42.9)											
At least some	32 (57.1)	0.58	0.34- 1.00	0.048								
Problem/s EQ-5D but no health condition												
No	56 (86.2)											
Yes	9 (13.9)	5	2.32-10.6	>0.001								

While specific health conditions were not included in the full analysis (because of small numbers), a higher proportion of LINKIN non-users reported drug or alcohol problems (10% versus 5% in users). Over half of the LINKIN non-users reported having bone and joint problems, while 25% reported having a heart condition / circulatory disease / high blood pressure. Given the small numbers of cases, these results should be interpreted with caution.

After adjusting for demographic and health-related characteristics, both datasets continued to show the same association with lack of health services use, with the exception of education, and the associations in the LINKIN data were consistently stronger. For the LINKIN dataset, non-users with fair or poor health, were more likely to be unemployed (aOR 3.24, 95% CI 1.28-8.17) smoke (aOR 2.67, 95% CI 1.38-5.17), be educated to Year 10 or below (aOR 1.94, 95% CI 1.02-3.70) and were less likely to report having a health condition (aOR 0.18, 95% CI 0.08-0.41) compared to users who reported fair or poor health (Table 2). The strength of the associations in the HILDA results were weaker due to wider confidence intervals, however the directions of the association were consistent with the LINKIN results. After adjustment, the association between non-users and educational level changed in the HILDA dataset for non-metropolitan regions to be consistent with findings from the adjusted LINKIN analysis (Table 2).

**Table 2:** Results from adjusted analysis comparing characteristics of non-users and users of health services who reported fair or poor health in the LINKIN and HILDA populations (Total and Non-metropolitan regions)

Characteristics	Adjusted LINKIN OR	95% CI	*P*	Adjusted Non Metro HILDA OR	95% CI	*P*	Adjusted Total HILDA OR	95% CI	*P*
Age	1.00	0.98-1.02	0.969	0.95	0.93-0.97	>0.001	0.99	0.97-1.01	0.323
Female	0.57	0.32-1.03	0.062	0.53	0.25-1.13	0.096	0.74	0.37-1.51	0.403
Single/never married	1.15	0.51-2.63	0.738	0.45	0.17-1.19	0.105	1.38	0.57-3.30	0.465
Educated: Year 10 or below	1.94	1.02-3.70	0.044	1.42	0.77-2.59	0.252	0.81	0.47-1.37	0.416
Unemployed	3.24	1.28-8.17	0.013	1.42	0.30-6.60	0.652	2.57	0.29-22.9	0.390
Smoker	2.67	1.38-5.17	0.004	2.16	0.94-4.96	0.068	1.59	0.66-3.83	0.294
1 or more health condition	0.18	0.08-0.41	<0.001	0.56	0.26-1.24	0.151	0.21	0.10-0.45	>0.001

Reference categories: Males; Married/living with partner/widowed; Highest education greater than Year 10; Not unemployed; Not current smoker; Does not self-report having a current health condition

## Discussion

This study has described a population which has remained out of view in Australian analyses and contemporary policy debate about health service utilisation. While this group constitutes a modest proportion of those with self-assessed fair or poor health (if extrapolated across Australia, the group approximates to 112,000 people aged 15 years or over), their lack of contact means that their health needs are likely to be poorly addressed, and in line with the ‘inverse care law’, they are likely to have greater health needs and poorer health outcomes [3, 18]. Specifically, people who self-rated their health as fair or poor and underused health services, were more likely unemployed and single. They were far more likely to smoke and tended to report at least moderate anxiety or depression. Many of these characteristics are indicators of social isolation and may be cumulative in a pathway of concentration of risk [19]. While the multivariable model did not indicate a significant p-value for gender and age, the 95% confidence intervals for the LINKIN data suggest that as shown with simple logistic regression, non-users tended to be younger and male. This pattern was supported by the HILDA data, and indeed, for those not living in a city, non-users were significantly likely to be younger in the multivariable model. This gender and age pattern of non-use is consistent with the findings of the recent international scoping review [7] which examined the characteristics of people not attending health checks. It should be noted that neither a meta-analysis nor meta-regression was conducted for this international review, an only a few studies included in the scoping review, included adjusted regression models.

Non-users were also less likely to report that they have at least one health condition. While this finding could be interpreted as indicating that there is no need for a health service visit, we should also consider that their very lack of contact with health services may mean that they were less likely to have formal diagnoses. While the numbers were small, it is notable that of those who did not report a health condition, non-users were more likely to report at least some problem on the EQ-5D. Also, given that non-users were more likely to have other risk factors, such as indicators of social isolation and smoking, this is precisely the group of people who have been identified as likely to benefit from anticipatory approaches to care [3, 4].

The high prevalence of smoking amongst non-users (from both the LINKIN and HILDA data) is an important finding. Given emerging recognition that there may be unforeseen consequences in public health anti-smoking initiatives which use ‘stigma’ and ‘de-normalising of smoking behaviour’ [20, 21] this strong association suggests that there may be barriers to providing stigma-free health care settings for people with health care needs and who smoke. Indeed, in line with Frohlich and Potvin arguments, this may be an example of how disparities in health can be exacerbated by population approaches [19, 22] and from an intervention perspective this ‘vulnerable population group’ may benefit from targeted approaches. This profile corresponds with findings of several qualitative studies focussed on underserved groups, such as a Canadian study where the reluctance to use health and social services by men living in poverty was examined. The research found that the nature of their problems (including internalising blame for their problems), a difficulty in seeking health (because of previous help seeking experiences and masculine norms) and the nature of services offered were key barriers to service use [23]. Another qualitative study which aimed to understand why people did not attend health checks in Britain found that previous experiences of primary care and a lack of personal relevance rather than lack of positive perception of the health check concept were barriers to attending a health check [24]. Both these studies and a recent review of how homeless people connect with community health and health promotion have emphasised the role of legitimate candidacy [25] in how people connect to health services. It is therefore important that future research takes account of both subjective perspectives on what makes people a legitimate candidate for health care [25] (including in-depth understandings of their experiences, values and priorities in managing their health) and points in the candidacy process which may be amenable to change [26] (related to practice, culture, structures) in order to improve connections with this population. An analysis of the specific effects of affordability and accessibility on use of health services was beyond the scope of this study.

This study has a number of important strengths, including the large population-based datasets, the range of variables covered, the inclusion of multivariable regression, and the administration of both surveys by trained personnel using a structured format. The cross-sectional study design of LINKIN limits this analysis to associations. It is also acknowledged that the self-completion nature of the questionnaire data in the LINKIN study may have led to some patient reporting error in recalling health service use, especially when the levels of non-use were low. However the consistency in the level of non-use across both data sources using different survey methods is reassuring. While the LINKIN dataset covered a single bounded regional location the consistency of results at a national level (HILDA dataset) supports the generalisability of these results. The generally weaker strengths of association in the HILDA results may reflect the complexity of the sample selection.

## Conclusion

By describing the distinct characteristics of this out-of-sight population this study improves understandings of the health needs of this group and highlights the risks tied to ‘lack of use’ becoming being a social determinant of health in its own right. For instance, in evaluations of public health campaigns the exclusion of the views and outcomes of non-users may mask important unintended consequences of such programs, such as further social isolation and non-use of services. Given the current recognition that evidence-based medicine needs to overcome biases tied to the way populations are identified, this study supports Greenhalgh et al’s [6] recent assertions related to the assessment of effectiveness of health policies and interventions, and specifically the need to expand our understandings to the real denominators in health need, and account for those who do not engage with the health system.

## Abbreviations

aOR: Adjusted odds ratio
DSS: Department of Social Services
EQ-5D-3L: EuroQol five dimensions three level questionnaire
HILDA: Household, Income and Labour Dynamics in Australia
OR: Odds ratio
SF-1: Short Form 1
SF-36: 6-Item Short Form Health Survey
